# Circulating vitamin D in relation to cancer incidence and survival of the head and neck and oesophagus in the EPIC cohort

**DOI:** 10.1038/srep36017

**Published:** 2016-11-04

**Authors:** Anouar Fanidi, David C. Muller, Øivind Midttun, Per Magne Ueland, Stein Emil Vollset, Caroline Relton, Paolo Vineis, Elisabete Weiderpass, Guri Skeie, Magritt Brustad, Domenico Palli, Rosario Tumino, Sara Grioni, Carlotta Sacerdote, H. B(as). Bueno-de-Mesquita, Petra H. Peeters, Marie-Christine Boutron-Ruault, Marina Kvaskoff, Claire Cadeau, José María Huerta, Maria-José Sánchez, Antonio Agudo, Cristina Lasheras, J. Ramón Quirós, Saioa Chamosa, Elio Riboli, Ruth C. Travis, Heather Ward, Neil Murphy, Kay-Tee Khaw, Antonia Trichopoulou, Pagona Lagiou, Eleni-Maria Papatesta, Heiner Boeing, Tilman Kuehn, Verena Katzke, Annika Steffen, Anders Johansson, Paul Brennan, Mattias Johansson

**Affiliations:** 1 International Agency for Research on Cancer, Lyon, France; 2 Bevital AS, Bergen, Norway; 3 Section of Pharmacology, Institute of Medicine, University of Bergen, Bergen, Norway; 4 Laboratory of Clinical Biochemistry, Haukeland University Hospital, Bergen, Norway; 5 Department of Public Health and Primary Health Care, University of Bergen, Bergen, Norway; 6 Division of Epidemiology, Norwegian Institute of Public Health, Bergen, Norway; 7 Institute for Ageing and Health, Newcastle University, Newcastle, United Kingdom; 8 School of Public Health, Imperial College London, London, United Kingdom; 9 HuGeF Foundation, Turin, Italy; 10 Department of Community Medicine, Faculty of Health Sciences, University of Tromsø, Tromsø, Norway; 11 The Arctic University of Norway, Tromsø, Norway; 12 Molecular and Nutritional Epidemiology Unit, Cancer Research and Prevention Institute – ISPO, Florence, Italy; 13 Cancer Registry and Histopathology Unit, “Civile M.P.Arezzo” Hospital, ASP Ragusa, Italy; 14 Epidemiology and Prevention Unit, Fondazione IRCCS Istituto Nazionale dei Tumori, Milan, Italy; 15 CPO-Piemonte and HuGeF Foundation, Torino Turin, Italy; 16 National Institute for Public Health and the Environment (RIVM), Bilthoven, The Netherlands; 17 Department of Gastroenterology and Hepatology, University Medical Centre, Utrecht, The Netherlands; 18 Department of Epidemiology, Julius Center for Health Sciences and Primary Care, University Medical Center, Utrecht, The Netherlands; 19 Nutrition, Hormones and Women’s Health team, Inserm, Centre for research in Epidemiology and Population Health (CESP), U1018, Villejuif, France; 20 Université Paris Sud, UMRS 1018, Villejuif, France; 21 Institut Gustave-Roussy (IGR), Villejuif, France; 22 Consortium for Biomedical Research in Epidemiology and Public Health (CIBER Epidemiología y Salud Pública-CIBERESP), Madrid, Spain; 23 Department of Epidemiology, Murcia Regional Health Council, Murcia, Spain; 24 Andalusian School of Public Health, Granada, Spain; 25 Catalan Institute of Oncology, L’Hospitalet de Llobregat, Spain; 26 Oviedo University, Oviedo, Spain; 27 Public Health Directorate Asturias, Oviedo, Spain; 28 Public Health Division of Gipuzkoa, BioDonostia Research Institute, Health Department of Basque Region, San Sebastian, Spain; 29 Cancer Epidemiology Unit, Nuffield Department of Clinical Medicine, University of Oxford, Oxford, United Kingdom; 30 School of Clinical Medicine, University of Cambridge, United Kingdom; 31 Hellenic Health Foundation, Athens, Greece; 32 WHO Collaborating Center for Food and Nutrition Policies, Department of Hygiene, Epidemiology and Medical Statistics, University of Athens Medical School, Athens, Greece; 33 Department of Epidemiology, Harvard School of Public Health, Boston, USA; 34 Bureau of Epidemiologic Research, Academy of Athens, Athens, Greece; 35 Department of Epidemiology, German Institute of Human Nutrition Potsdam-Rehbruecke, Nuthetal, Germany; 36 German Cancer Research Center DKFZ, Heidelberg, Germany; 37 Nutritrional Research/Molecular Periodontology Umeå University, Umeå, Sweden

## Abstract

Experimental and epidemiological data suggest that vitamin D play a role in pathogenesis and progression of cancer, but prospective data on head and neck cancer (HNC) and oesophagus cancer are limited. The European Prospective Investigation into Cancer and Nutrition (EPIC) study recruited 385,747 participants with blood samples between 1992 and 2000. This analysis includes 497 case-control pairs of the head and neck and oesophagus, as well as 443 additional controls. Circulating 25(OH)D_3_ were measured in pre-diagnostic samples and evaluated in relation to HNC and oesophagus cancer risk and post-diagnosis all-cause mortality. After controlling for risk factors, a doubling of 25(OH)D_3_ was associated with 30% lower odds of HNC (OR 0.70, 95% confidence interval [95% CI] 0.56–0.88, *P*_*trend*_ = 0.001). Subsequent analyses by anatomical sub-site indicated clear inverse associations with risk of larynx and hypopharynx cancer combined (OR 0.55, 95CI% 0.39–0.78) and oral cavity cancer (OR 0.60, 95CI% 0.42–0.87). Low 25(OH)D_3_ concentrations were also associated with higher risk of death from any cause among HNC cases. No clear association was seen with risk or survival for oesophageal cancer. Study participants with elevated circulating concentrations of 25(OH)D_3_ had decreased risk of HNC, as well as improved survival following diagnosis.

About 650,000 new cases of head and neck cancer (HNC) and 450,000 new cases of esophageal cancer occur worldwide each year^1^. This corresponds to approximately 11% of total cancer incidence. Global differences in incidence are primarily driven by its main risk factors tobacco and alcohol exposure^2^,^3^, but over the last decade infection by human papillomavirus has emerged as an important cause of HNC subsites, in particular oropharynx cancer^4^.

Vitamin D is a fat-soluble precursor to the steroid hormone calcitriol, primarily obtained via endogenous synthesis in the skin after exposure to sunlight^5^,^6^, and is fundamental for absorption of dietary calcium and maintaining bone health^5^,^7^. Vitamin D is also thought to have a protective role in cancer development and progression, and there is a large body of evidence from mechanistic studies supporting a role of vitamin D in multiple hallmarks of carcinogenesis^7^,^8^.

Vitamin D is metabolised in the liver to 25-hydroxyvitamin D [25(OH)D] and can be measured in the circulation to assess an individual’s vitamin D status^8^. In contrast to mechanistic studies, epidemiological studies of circulating 25(OH)D have not provided consistent evidence supporting a beneficial role of vitamin D in terms of cancer incidence, the exception being colorectal cancer for which the majority of studies support an inverse association between circulating vitamin D and risk^9^,^10^. Circulating 25(OH)D has not been frequently studied in relation to cancers of the head and neck and esophagus, and the literature is to date limited and typically total based on measures of total vitamin D (25(OH)D). One small Danish study based on 44 HNC cases from three cohorts found a nominal inverse association between circulating 25(OH)D and HNC risk^11^. From larger studies of circulating 25(OH)D and HNC risk, there is evidence both of an inverse association with risk, and of no association. The Copenhagen City Heart Study (CCHS) based on 122 HNC cases reported a 45% increase hazards of HNC for a 50% reduction in circulating vitamin D^12^ whereas the Alpha-Tocopherol Beta Carotene (ATBC) study based on 340 HNC cases did not observe any clear association between circulating vitamin D and HNC risk^13^. Furthermore, the Vitamin D Pooling Project (VDPP) combining 8 prospective cohorts and based on 267 esophageal cancer cases did not find any association with risk^14^. In addition, none of these studies have evaluated whether pre-diagnostic 25(OH)D is also related to risk of death after diagnosis.

While reverse causality is a principal concern in retrospective studies, the current study aimed to provide a comprehensive evaluation of pre-diagnostic circulating 25(OH)D and risk of cancers of the head and neck and oesophagus from a large case-control study nested within the European Prospective Investigation into Cancer and Nutrition (EPIC). In addition, we aimed to evaluate if pre-diagnostic circulating 25(OH)D is associated with survival following cancer diagnosis.

## Results

### Baseline characteristics

The final study population consisted of 497 cases, including 350 head and neck cancers and 147 oesophagus cancers, 497 individually matched controls and 443 subjects in control group 2. Approximately two thirds (68%) of the nested case-control population were men (Table 1), the median age at recruitment was 56.7 years (5^th^–95^th^ percentile: 42–71) and the average time from blood draw to diagnosis for cases was 6.3 years. Control group 2 had similar demographic characteristics as the matched control group, but with a higher proportion of women.

### Variations in circulating 25(OH)D_3_ by season and demographic characteristics

**Figure 1:**
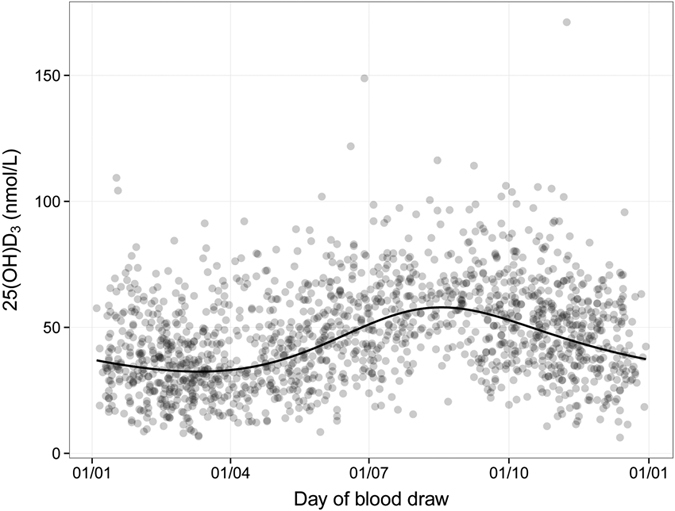
Seasonal variation of 25(OH) D_3_ concentrations among all study participants. Scattered points show the measured values. The solid line represents the predicted geometric mean concentration given day of blood draw, which was modelled as a linear combination of sine and cosine functions. See the text of the methods section for further details.

Predicted and observed concentrations in relation to season of blood draw are displayed in Fig. 1. Visual inspection suggests clear differences in circulating 25(OH)D_3_ that are consistent with sun exposure being the most important determinant of circulating vitamin D, 25(OH)D_3_ concentrations were lowest among those who had their blood drawn around February and March and highest among those who had their blood drawn around August.

The relation of nutrient intake, lifestyle factors, smoking, and alcohol intake with plasma 25(OH)D_3_ are presented in Supplementary Table 1. In comparison with control participants who reported being never smokers, current smokers had 7% lower 25(OH)D_3_ concentrations on average (95% CI: −14% to 0%), whereas former smokers had 9% higher 25(OH)D_3_ concentrations (95% CI: 2–16%). BMI was inversely associated with 25(OH)D_3_ concentrations. No differences in 25(OH)D_3_ were seen in relation to alcohol intake.

### Circulating 25(OH)D_3_ in relation to head and neck and esophagus cancer risk

The risk analysis results for overall HNC overall are shown in Table 2. Participants with higher 25(OH)D_3_ concentrations had lower HNC risk, with a doubling in plasma 25(OH)D_3_ being associated with 45% lower risk (conditional OR_log2_ 0.55, 95% CI 0.42–0.72, *P*_*trend*_ 1 × 10^−5^; unconditional OR_log2_ 0.54, 95% CI 0.44–0.68, *P*_*trend*_ 3 × 10^−8^). Following adjustments for education, alcohol consumption, circulating cotinine, tobacco exposure, and BMI, the unconditional OR estimates were slightly attenuated but remained indicative of an inverse association between circulating 25(OH)D_3_ and risk (OR_log2_0.70, 95% CI 0.56–0.88, *P*_*trend*_ 0.001). We also evaluated the association between concentrations of 25(OH)D_3_ and oesophageal cancer squamous cell carcinoma and adenocarcinoma, and whilst the OR estimates were consistent with that of overall HNC, the confidence intervals were wide (OR 0.69, 95% CI 0.44–1.10 for esophageal squamous cell carcinoma [ESCC], and OR 0.74, 95% CI 0.45–1.23 for esopheageal adenocarcinoma [EADC]). Because one of the key functions of vitamin D is to maintain calcium homoeostasis, we also included calcium intake in our models, but this did not affect the OR estimates (data not shown).

**Figure 2:**
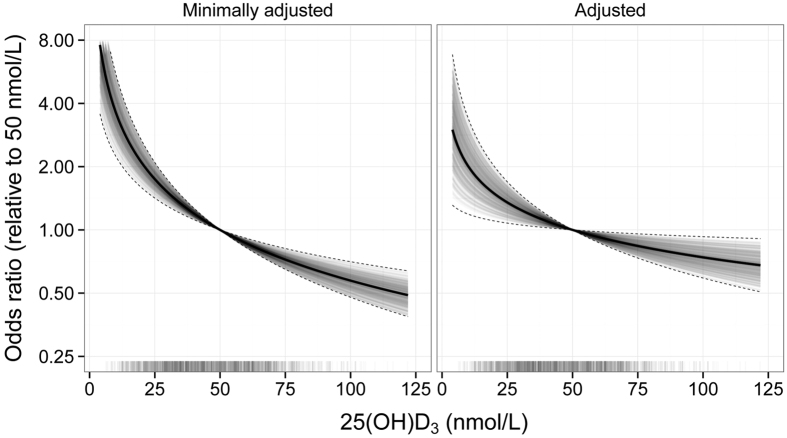
Odds ratio for head and neck cancer as a function of circulating concentrations of 25(OH) D_3_, relative to a concentration of 50 nmol/L. Log-base-2 25(OH) D3 was modelled as a continuous covariate. The left panel shows the minimally adjusted estimate, adjusted for age at recruitment (in 5 year groups), sex, country, and seasonality (sine and cosine functions of day of blood draw). The right panel depicts the association after additional adjustment for educational attainment (in 5 groups), smoking status (never/former/current/missing), circulating cotinine (quartiles defined among the current smokers), alcohol intake at recruitment (g/day), and BMI (in 3 groups). Solid and dashed lines represent the maximum likelihood estimates and 95% confidence intervals respectively. The translucent lines are 1000 draws from the multivariate normal distribution defined by the maximum likelihood estimates and their variance covariance matrix, and thus give an indication of the posterior density for the odds ratio under a uniform prior on the regression coefficients. The “rug plot” under each panel shows the observed distribution of 25(OH) D3.

ORs of HNC across the observed range of 25(OH)D_3_ concentrations are depicted in Fig. 2. Compared to participants having 25(OH)D_3_ blood concentrations of 50 nmol/L, odds of HNC were 42% higher for those with 25(OH)D_3_ blood concentrations of 25 nmol/L, and 30% lower for those with 25(OH)D_3_ blood concentrations of 100 nmol/L.

### Circulating 25(OH)D_3_ risk analysis stratified for head and neck cancer sub-sites demographic and lifestyle factors

Risk analyses stratified by HNC sub-sites are displayed in Table 3, and indicated that the association of 25(OH)D_3_ was particularly prominent for cancers of the larynx and hypopharynx (OR 0.55, 95% CI 0.39–0.78) and for cancers of the oral cavity (OR 0.60, 95% CI 0.42–0.87). No association was observed with risk of oropharynx cancer (OR 0.92, 95% CI 0.58–1.45), although the sample size was small.

Stratifications by demographic and lifestyle factors (Supplementary Figure 1) showed that the association between circulating 25(OH)D_3_ and HNC risk was solely driven by former (unadjusted OR_log2_ 0.65, 95% CI 0.42–1.01) and current smokers (unadjusted OR_log2_ 0.47, 95% CI 0.34–0.66), and was not seen in never smokers (OR_log2_ 1.21, 95% CI 0.72–2.03; (*P*_*heterogeneity*_ 0.02). In order to further evaluate if residual confounding by tobacco exposure might explain the association between circulating 25(OH)D_3_ and HNC risk in current smokers, we adjusted for circulating cotinine–a useful and objective indicator of recent tobacco exposure–which resulted in a small attenuation in OR estimates (cotinine adjusted OR_log2_ 0.55, 95% CI 0.39–0.80). Additional indicators of historical tobacco exposure, in particular duration and average number of cigarettes smoked per day (CPD), were incomplete and only available for 85% of the study population. Excluding subjects with missing data on these indicators yielded unadjusted OR estimates of 0.71 in former smokers (95% CI 0.44–1.14) and 0.46 (95% CI 0.31–0.66) in current smokers, with small attenuations in OR being observed when additionally adjusting for duration and CPD (former smokers OR_log2_ 0.77, 95% CI 0.45–1.24, current smoker OR_log2_ 0.55, 95% CI 0.36–0.81).

We also noted that the inverse association of circulating 25(OH)D_3_ with HNC risk was not affected after excluding cases within one year and 2 years after blood draw (OR_log2_ 0.69, 95% CI 0.54–0.88 and 0.69, 95% CI 0.54–0.89; respectively). Furthermore, the inverse association between circulating 25(OH)D_3_ and HNC risk was similar for cases diagnosed within three years after blood draw, as well as for those being diagnosed over 9 years after their blood draw (P for heterogeneity 0.79). Some indications of differences in strength of association between circulating 25(OH)D_3_ and HNC risk were also seen when stratifying for groups of alcohol intake at recruitment, where no association was seen among subject with 0 grams of alcohol intake per day, although OR estimates in the other drinking categories were compatible with that overall.

### All-cause mortality for study participants diagnosed with HNC and oesophagus cancer

In total, 145 deaths occurred among the HNC cases during the follow-up. Median time between diagnosis and death for those cases who died during follow-up was 15 months (range: 0 months to 12 years), and median time between blood collection and death was 8.5 years (range: 7 months to 15 years).

**Figure 3:**
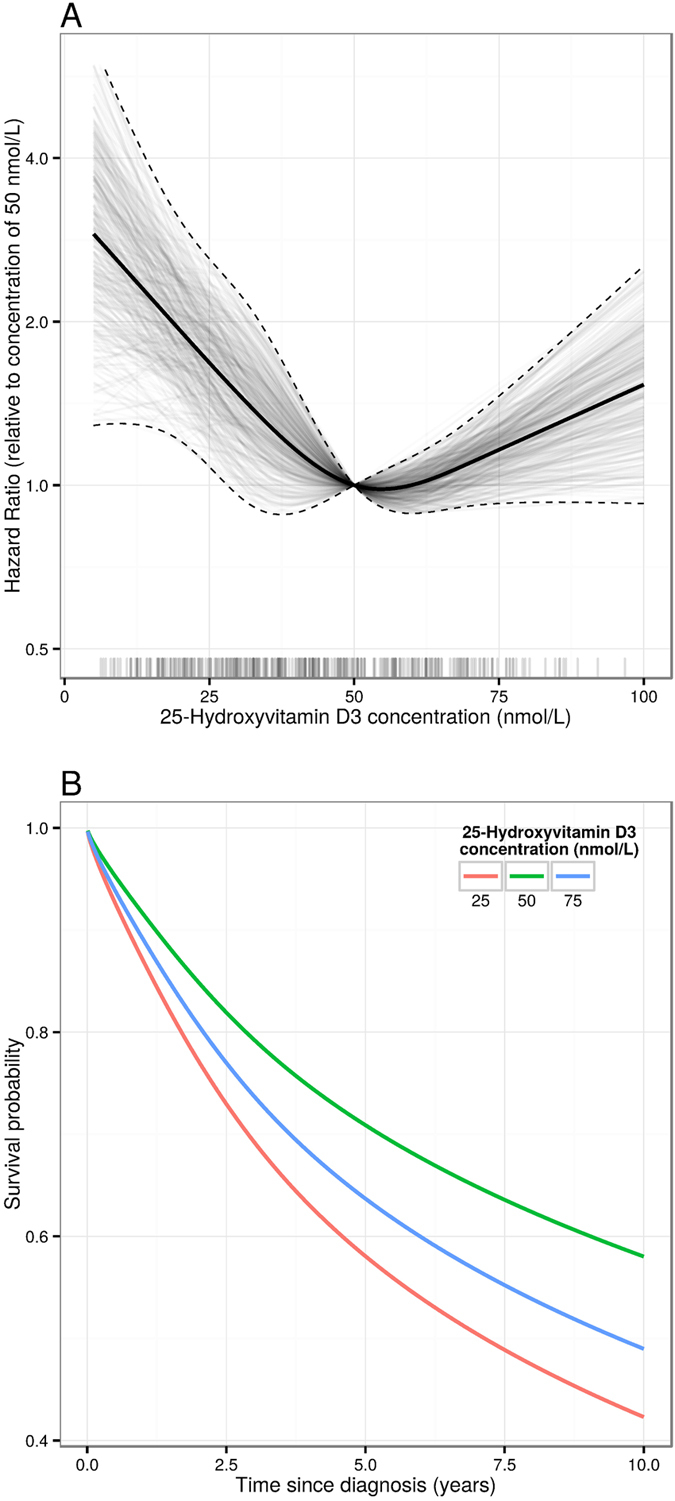
Post head and neck cancer survival. Panel A: Hazard ratio from a Cox model for all-cause mortality post HNC diagnosis as a function of circulating concentration of 25(OH) D_3_, relative to a concentration of 50 nmol/L. 25(OH) D_3_ was modelled using restricted cubic splines with knots at the 10th, 33rd, 67th, and 90th percentiles of its distribution. The model was adjusted for age at recruitment (in 5 year groups), sex, country, seasonality (sine and cosine functions of day of blood draw), educational attainment (in 5 groups), smoking status (never/former/current/missing), circulating cotinine (quartiles defined among the current smokers) alcohol intake at recruitment (g/day), and BMI (in 3 groups). Solid and dashed lines represent the maximum likelihood estimates and 95% confidence intervals respectively. The translucent lines are 1000 draws from the multivariate normal distribution defined by the maximum likelihood estimates and their variance covariance matrix, and thus give an indication of the posterior density for the hazard ratio under a uniform prior on the regression coefficients. The “rug plot” shows the observed distribution of 25(OH) D_3_. Panel B: Survival function post HNC diagnosis evaluated at given concentrations of 25(OH) D_3_, derived from a flexible parametric survival model. Restricted cubic splines with knots at the 0th, 33rd, 67th, and 100th percentiles of the distribution of uncensored survival times were used to model the baseline hazard. Like the Cox model used to derive panel A, 25(OH) D_3_ was modelled using restricted cubic splines with knots at the 10th, 33rd, 67th, and 90th percentiles of its distribution.

Results of Cox proportional hazards regression for 25(OH)D_3_ of all-cause mortality are shown in Fig. 3A. Similarly to the analysis of incidence, an overall inverse association between pre-diagnostic circulating 25(OH)D_3_ and survival post HNC was observed; the HR for log_2_ 25(OH)D_3_ [HR_log2_] being 0.73 (95% CI 0.55–0.97). The hazard of death was 1.72 times higher (95% CI: 1.11–2.51) for participants with circulating concentrations of 25 nmol/L compared to those with 50 nmol/L. However, no further survival benefits were seen for cases with 25(OH)D_3_ concentrations above 50 nmol/L, and there was even an indication that higher 25(OH)D_3_ concentrations might be associated with elevated hazard of death, although very few participants had 25(OH)D_3_ greater than 75 nmol/L. For those deceased with HNC specifically indicated as underlying cause of death (87 deaths), HR_log2_ was 0.68 (95% CI 0.51–0.91) and adjusting the cause-specific mortality analysis for potential confounders did not affect the HR estimates notably (HR_log2_ 0.71, 95% CI 0.53–0.96, *P*_*trend*_ = 0.02). For comparison, 58 deaths occurred with other-than-HNC indicated as cause of death, and no association between circulating 25(OH)D_3_ and survival was observed, the HR_log2_ being 0.88 (95% CI = 0.53–1.26, *P*_*trend*_ = 0.29).

Model-based estimates of the survival function evaluated at 25, 50, and 75 nmol/L of 25(OH)D_3_ among HNC cases are presented Fig. 3B. The expected 5-year post diagnostic survival probabilities for cases with 25(OH)D_3_ concentrations of 25 nmol/L were 0.58 (95% CI 0.49, 0.65), and 0.71 (95% CI 0.64, 0.77) for those having 25(OH)D_3_ concentrations of 50 nmol/L. Estimated HRs did not vary notably when stratifying by sex, age, country, education, alcohol or tobacco exposure (Supplementary Figure 2). Of note, when stratifying the survival analysis by time from blood draw to diagnosis, the association of circulating 25(OH)D_3_ with all-cause mortality was apparent both among HNC cases diagnosed before 5 years (HR_log2_ 0.72, 95% CI 0.54–0.95) and those diagnosed over 5 years after blood draw (HR_log2_ 0.80, 95% CI 0.62–1.03). Regarding the 91 death that occurred among esophageal cancer, circulating concentrations of 25(OH)D_3_ did not display any clear association with all-cause mortality, nor with esophageal-specific cause mortality.

We had limited information on disease stage, and adjusting for stage in an analysis restricted to those 161 HNC cases (46%) for whom stage was available appeared to completely attenuate the association of circulating 25(OH)D_3_ with all-cause mortality (HR_log2_ = 0.96 95% CI = 0.76–1.20, *P*_*trend*_ = 0.64), suggesting that the inverse association with all-cause mortality noted in the unadjusted survival analysis was completely mediated by difference in disease stage.

## Discussion

To date, this is the largest prospective study investigating the relation between circulating vitamin D and head and neck cancer risk, and the first to also evaluate the relation with post-diagnosis survival. We report a notable risk decrease for participants with higher plasma vitamin D concentrations, as well as improved survival among cases having adequate pre-diagnostic vitamin D concentrations.

### Vitamin D in cancer development and progression

The importance of vitamin D in maintaining bone health has long been recognized, and there is also an abundance of mechanistic studies supporting a beneficial role of vitamin D in carcinogenesis by inhibiting tumour initiation and progression. Specifically, circulating vitamin D (measured as 25(OH)D_3_ in the current study) is a precursor to its active hydroxylated form calcitriol (1,25(OH)D_3_). Calcitriol is a potent steroid hormone that has been implicated by *in vitro* and *in vivo* models as having a potential anti-cancer influence by affecting multiple cancer hallmarks, including reducing angiogenesis, metastasis, cell invasion, inflammation, and proliferation, as well as stimulating apoptosis^7^. Some studies have also demonstrated a beneficial role of calcitriol in tobacco-related cancers, including oral, lung and bladder cancer, where reductions in angiogenesis, metastasis, and cell-modulatory effects have been suggested^7^,^8^,^15^,^16^,^17^,^18^,^19^,^20^.

### Prospective studies of 25(OH)D_3_ and head and neck cancer

Circulating vitamin D has been widely studied in relation to risk of multiple cancers in prospective studies, with the most consistent evidence accumulated to date being an inverse association of colorectal cancer^9^,^10^. The body of evidence on other cancers is contradictory and does not support a similar consistent inverse association of vitamin D with other common or rare cancers^7^. Head and neck cancer has been rarely studied in relation to circulating vitamin D, with only three prospective studies published to date; the CCHS based on 122 cases^12^, the ATBC study based on 340 cases^13^, and an additional small Danish study based on 44 cases from three cohorts^11^. The ATBC study did not observe any association between 25(OH)D and head and neck cancer risk, nor did the smaller Danish study, although the latter was underpowered to detect any notable association with risk. This contrasts to the results of the CCHS study which was a classical cohort study that assessed baseline 25(OH)D for a complete cohort of 9,791 participants at baseline, and then followed them for up to 28 years for a wide range of incident cancers. The CCHS study conducted their analysis by estimating the relative risk associated with a 50% reduction in circulating vitamin D (OR_1/2_), and whilst no association was noted for non-smoking related cancers overall (OR_1/2_: 0.95), the CCHS study reported a clear inverse association between 25(OH)D and smoking related cancers and circulating vitamin D (OR_1/2_: 1.20), with a particularly strong association for HNC (OR_1/2_: 1.44). The result was almost identical to that of the current EPIC study were we observed an OR of 0.70 for a doubling in 25(OH)D_3_, corresponding to an OR_1/2_ of 1.45. The VDPP study, a consortium of 8 prospective cohorts including 143 ESCC and 100 EADC, did not observe any clear relation with circulating vitamin D, an observation similar to our results.

We note that in the current study, the inverse association between vitamin D and HNC risk was solely observed in former and current smokers. In accordance with results from the vitamin D pooling project^21^, we observed 8% lower circulating vitamin D concentrations in current smokers compared to never smokers, but concurrently 8% higher circulating vitamin D in former smokers compare to never smokers (Supplementary Table 1). Residual confounding by tobacco exposure is therefore an important concern when interpreting the results, in particular among current smokers. In smoking stratified analyses we adjusted for several indicators of tobacco exposure, included circulating cotinine. Given that the inverse associations with risk in former and current smokers were largely unchanged after adjusting for these additional tobacco exposure indicators, residual confounding by tobacco exposure does not appear to explain the observed decreases in head and neck cancer risk among study participants with elevated circulating vitamin D.

Further, the observed association was very stable during the follow-up, and most notable for a follow-up time of 9 years after recruitment, suggesting that the findings are unlikely to be due to reverse causation (Supplementary Figure 1). Considering that we were able to carefully control for current tobacco exposure using circulating cotinine measures, as well as alcohol consumption, we therefore interpret these results to mean that the decrease in head and neck cancer risk observed with elevated circulating vitamin D cannot be readily explained by known risk factors.

### Vitamin D and cancer progression

Head and neck cancer prognosis is generally poor with overall 5-year survival rates of 50% to 60%, an important reason being that a large fraction of cases are diagnosed at advanced disease stage^22^,^23^,^24^. It was therefore interesting to note that in our analysis of all-cause mortality, pre-diagnostic 25(OH)D_3_ was inversely associated with survival among head and neck cancer cases following their diagnosis. In contrast to the association noted with HNC risk, for which a log-linear trend was observed across the whole range of observed 25(OH)D_3_ concentrations, the association with survival was only evident in the lower 25(OH)D_3_ range. This manifested in 72% higher hazard of death for cases with 25(OH)D_3_ concentrations of 25 nmol/L compared with those with 50 nmol/L, with corresponding 5-year survival probabilities of 58% and 71%, respectively. Estimated HRs did not vary notably when stratifying by time from blood draw to diagnosis, suggesting that pre-clinical symptoms would not seem likely to explain the association with all-cause mortality. Furthermore, subsequent cause-specific survival indicated that the association of 25(OH)D_3_ was primarily driven by deaths caused by HNC, but not other causes of death. However, taking disease stage into account appeared to explain most of the survival association of vitamin D, indicating that any survival benefit of having adequate pre-diagnostic 25(OH)D levels for HNC cases is likely to be mediated through differences in disease stage at diagnosis. Furthermore, it would have been informative to attain blood samples at diagnosis to fully evaluate the importance of circulating vitamin D for HNC prognosis.

### Advantages and limitations

Our study distinguished itself from most previous prospective studies on vitamin D in that we used a mass spectrometry-based platform (LC-MS/MS) to specifically assay 25(OH)D_3_, rather than the more commonly used enzyme-linked immunosorbent assay (ELISA) which measures 25(OH)D_2_ and 25(OH)D_3_ combined. Because the vast majority of total circulating vitamin D is in the form of 25(OH)D_3_, we would not expect this to explain any discrepant results with other studies, even though evaluating 25(OH)D_3_ would seem preferable as it precedes the most active form of vitamin D, calcitriol (1,25(OH)D_3_)^7^. Another distinguishing factor of our analysis was that we controlled for seasonal variations in vitamin D using trigonometric functions (Fig. 1), rather than discontinuous seasonal models, such as discrete variables indicating the four seasons. Using trigonometric functions has an advantage in providing a smooth seasonal model with minimum degrees of freedom, whilst providing a valid account of the inherent differences in vitamin D related to sun exposure. Still, given the prospective design of our study, we do not expect differential bias between cases and controls by season, and the most important distinguishing factors comparing ours and others studies on circulating vitamin D in relation to HNC risk are *a)* the large study population and *b)*, the multicentre recruitment across multiple European countries which would make the results more generalizable to other populations.

The principal limitation of our study is that 25(OH)D_3_ was measured using a single blood sample drawn in adulthood and therefore it may be possible that a single measurement does not capture exposure to vitamin D in an etiologically relevant period. Furthermore, in risk analyses stratified by cancer site, as well as in analyses of oesophagus cancer subtypes, the resulting small sample size hampered the statistical power to provide accurate risk estimates of potential risk associations.

## Conclusions

EPIC participants with adequate circulating concentrations of vitamin D had lower risk of head and neck cancer and improved survival following their diagnosis. Known risk factors could only partly explain these associations. Overall, our results are consistent with a beneficial role of vitamin D in head and neck cancer aetiology.

## Materials and Methods

### Study cohort

The study was conducted within the European Prospective Investigation into Cancer and nutrition (EPIC). EPIC recruitment procedures, collection of questionnaire data, anthropometric measurements, and blood samples have been described in detail elsewhere^25^. In brief, 521,330 individuals were recruited between 1992 and 2000 by 23 centres across 10 European countries, of which 385,747 contributed a blood sample. Blood fractions were aliquoted into 0.5 mL straws, which were heat-sealed and stored in liquid nitrogen tanks at −196 °C, except at the Umeå centre in Sweden where samples were stored in 1.8 mL plastic tubes in −80 °C freezers. Participants completed self-administered questionnaires on lifestyle factors and diet.

### Follow-up for Cancer Incidence

Incident cancer cases were identified at regular intervals through population-based cancer registries (Denmark, Italy except Naples, the Netherlands, Norway, Spain, Sweden and United Kingdom) or by active follow-up (France, Germany, Greece and Naples), which involved a combination of methods, including review of health insurance records, cancer and pathology registries, as well as direct contact with participants and their next-of-kin.

Mortality data, including vital status, cause and date of death, were obtained from mortality registries at the regional or national level. Subjects were followed up from study entry until cancer diagnosis (except non-melanoma skin cancer), death, emigration, or the end of the follow-up period for the relevant study centre. End of follow-up was defined as the latest date of complete follow-up for both cancer incidence and vital status and varied between study centres from December 2004 to June 2010. Vital status at follow-up is over 98% complete.

### Selection of Case and Control Participants

We initially identified 1,273 subjects diagnosed with incident head and neck or oesophagus cancer within the entire EPIC cohort by the end of the follow-up period for all centres. These cancer cases were defined on the basis of the *International Classification of Diseases for Oncology, Second Edition* (ICD-O-2), and included: oral cavity (ICD C02.0-C02.9, C04.0-C04.9, C03.0-C03.9, C05.0-C06.9, C14.0-C14.9), oropharynx (C01.9, C02.4, C09.0-C10.9), hypopharynx (C13.0-C13.9), larynx (C32.0-C32.9), and oesophagus (C15.0-C15.9). Cases who did not donate a blood sample (n = 152), did not have enough plasma available for biochemical analysis (n = 20), had a history of another cancer (n = 158, except non melanoma skin cancer), were not histologically confirmed, or did not have questionnaire information available (n = 22), were excluded, leaving 921 eligible cases. Because the aetiology of squamous cell carcinoma (SCC) and adenocarcinoma are thought to differ and the vast majority of HNC are SCC, we focused our analysis on SCC by excluding adenocarcinoma of the head and neck (n = 16). Also, we excluded esopheageal cases that were not classified as SCC or adenocarcinoma (n = 19). After further excluding cases from Denmark (n = 288) and the Malmö centre in Sweden (n = 101) who did not participate in this study, 497 eligible case participants with plasma samples were available for biochemical analyses. Data on histology were collected from each centre where possible.

For each case participant, one control was randomly chosen from appropriate risk sets consisting of all cohort members alive and free of cancer (except non-melanoma skin cancer) at the time (and hence age) of diagnosis of the index case. Matching criteria were country, sex, date of blood collection (±1 month, which was relaxed to ± 5 months for 22% of sets without available controls), and date of birth (±1 year, which was relaxed to ± 5 years for 1% of sets without available controls). In addition, we included 443 additional controls (control group 2) that were analysed in the context of a parallel study and individually matched to cases of another cancer site using identical matching criteria^26^.

The final dataset included 497 cancer cases and 497 individually matched controls, as well as 443 additional unmatched controls from control group 2 that contributed to unconditional and stratified risk analyses.

This research was conducted according to the principles expressed in the Declaration of Helsinki. This study was approved by the ethics review boards of the International Agency for Research on Cancer and individual EPIC centres. Informed consent was obtained from all EPIC participants at recruitment for use of their blood samples and data in future research.

### Biochemical Analyses

All biochemical analyses were performed at Bevital A/S (http://www.bevital.no), Bergen, Norway. Liquid chromatography coupled to tandem mass spectrometry was used to measure plasma concentrations of 25-hydroxyvitamin D_2_ [25(OH)D_2_] and 25-hydroxyvitamin D_3_ [25(OH)D_3_]^27^, as well as cotinine^27^ (as indicator of recent nicotine exposure). We found that 25(OH)D_2_ was undetectable in the majority of the samples, thus our analyses focus on 25(OH)D_3_. We performed sensitivity analyses by using the sum of 25(OH)D_3_ and 25(OH)D_2_ (setting undetectable levels of 25(OH)D_2_ to 0) and we observed similar results.

Samples were analysed in batches of 86 and quality control included 6 calibration samples, 2 control samples, and 1 blank sample in each batch. The limit of detection for 25(OH)D_3_ was 6.3 nmol/L, and within- and between-day coefficients of variation were 4.4 to 8.2%. All plasma samples were kept at −80 °C and all HNC and oesophagus cancer cases and their individual matched controls were analysed together within the same batches in random order. Samples from control group 2 were evenly and randomly interspersed across the batches to ensure that no differential differences in 25(OH)D concentrations occurred. The laboratory staff was blinded to the case-control status of the blood samples.

### Statistical Analyses

We initially used conditional logistic regression, conditioning on matched case-sets, to evaluate the relation between circulating 25(OH)D_3_ and disease risk. Odds ratios (OR) and 95% confidence interval (CI) were calculated for the base 2 logarithm (log2) of 25(OH)D_3_ as estimates of the relative risk associated with a doubling in 25(OH)D_3_ concentrations. In order to increase the statistical power, we incorporated control group 2 in the risk analysis by use of unconditional logistic regression, adjusting for age at recruitment in years, sex and country of recruitment, as well as seasonality in 25(OH)D_3_ concentrations by fitting two pairs of sine and cosine functions of the day of blood draw (scaled to be between 0 and 1 through out any year). This method efficiently accounts for seasonal effects by providing smooth predictions without artificial discontinuities throughout the year. The logistic regression estimates were nearly identical with and without control group 2, and we therefore focused the result presentation of the risk analysis on the unconditional analyses with the matched controls and control group 2 combined. Additional adjustments were performed to account for possible confounding by known risk factors, including tobacco exposure (smoking status [never, former, current], and for cotinine concentrations [defined using quantiles of the distribution among current smokers]), alcohol consumption (self-reported intake in grams per day at recruitment [g/day]), as well as educational attainment (in five categories). Including additional smoking variables (duration of smoking, average cigarettes smoked per day) and body mass index did not alter the results notably and were not included in the final models. In order to the assess the statistical significance, log-linear trends (*P*_*trend*_) were calculated by including the base 2 logarithm (log2) of the biomarker concentration as a continuous variable in separate logistic regression models.

The relation between circulating pre-diagnostic 25(OH)D_3_ and post-diagnostic survival was assessed by calculating hazard ratios (HR) of all-cause mortality among incident head and neck and oesophagus cancer cases using a Cox proportional hazards model with time since diagnosis as the time-scale. We modelled 25(OH)D_3_ using restricted cubic splines with knots at its 10th, 33rd, 67th, and 90th percentiles, adjusting for the same covariates as in the unconditional logistic regression models, as well as age at diagnosis (years). Visual inspection of smoothed scaled Schoenfeld residuals revealed no notable departure from proportional hazards. In order to estimate the survival function at given concentrations of 25(OH)D_3_, we fitted a flexible parametric survival model^28^, modelling the baseline cumulative hazard with restricted cubic splines (knots at the 0th, 33rd, 67th, and 100th percentiles of the distribution of failure times).

The statistical uncertainty was evaluated by sampling from the asymptotic distribution of the regression coefficients (the multivariate normal distribution with location and scale given by the maximum likelihood estimates and their variance-covariance matrix respectively). 1000 samples were drawn for each model and used to generate plausible predicted OR and HR. We plotted predictions that fall within the 95% CI to provide a visual impression of the 95% highest posterior density for the estimates under uniform prior distributions. All P-values were two-sided and statistical analyses were conducted using SAS version 9.2 (Cary, NC) and R version 3.0.2^29^.

### Ethical approval

This study was approved by the ethics review boards of the International Agency for Research on Cancer and individual EPIC centres. All EPIC participants provided written consent at recruitment for use of their blood samples and data in future research.

## Additional Information

**How to cite this article**: Fanidi, A. *et al*. Circulating vitamin D in relation to cancer incidence and survival of the head and neck and oesophagus in the EPIC cohort. *Sci. Rep.* **6**, 36017; doi: 10.1038/srep36017 (2016).

**Publisher’s note:** Springer Nature remains neutral with regard to jurisdictional claims in published maps and institutional affiliations.

## Supplementary Material

Supplementary Information

## Figures and Tables

**Table 1:** Baseline and Clinical Characteristics of Study Participants.

Discrete variables	No. (%) of Participants in Group		
	Cases (n = 497)	Matched controls (n = 497)	Control group n°2 (n = 443)
Participating countries			
France	5 (1%)	5 (1%)	13 (3%)
Italy	63 (13%)	63 (13%)	88 (20%)
Spain	91 (18%)	91 (18%)	52 (12%)
United Kingdom	117 (24%)	117 (24%)	67 (15%)
The Netherlands	70 (14%)	70 (14%)	46 (10%)
Greece	20 (4%)	20 (4%)	17 (4%)
Germany	96 (19%)	96 (19%)	124 (28%)
Sweden	34 (7%)	34 (7%)	32 (7%)
Norway	1 (0%)	1 (0%)	4 (1%)
Sex			
Men	340 (68%)	340 (68%)	234 (53%)
Women	157 (32%)	157 (32%)	209 (47%)
Smoking status			
Never	103 (21%)	206 (41%)	206 (47%)
Former	137 (28%)	177 (36%)	144 (33%)
Years since quitting <10	51(39)	46 (27)	40 (28)
Years since quitting ≥ 10	80 (61)	125 (73)	102 (72)
Current	249 (50%)	101 (20%)	90 (20%)
Unknown	8 (2%)	13 (3%)	3 (1%)
Education			
Primary school or less	219 (44%)	192 (39%)	171 (39%)
Technical/professional school	113 (23%)	132 (27%)	100 (23%)
Secondary school	75 (15%)	61 (12%)	58 (13%)
Higher education	68 (14%)	94 (19%)	100 (23%)
Unknown	22 (4%)	18 (4%)	14 (3%)
Alcohol intake at recruitment (g/d)			
= 0	86 (17%)	58 (12%)	54 (12%)
0.1–6	133 (27%)	162 (33%)	161 (36%)
6.1–12	45 (9%)	74 (15%)	55 (12%)
12.1–24	66 (13%)	88 (18%)	82 (19%)
24.1–60	107 (22%)	90 (18%)	74 (17%)
60.1–96 in men or >60 in women	43 (9%)	18 (4%)	14 (3%)
>96 in men	15 (3%)	7 (1%)	3 (1%)
Body Mass Index (BMI)^a^			
<25	195 (39%)	197 (40%)	179 (40%)
25–29.9	221 (44%)	231 (46%)	187 (42%)
≥ 30	81 (16%)	69 (14%)	77 (17%)
**Continuous variables, median (5^th^–95^th^ percentile)**			
Age at blood draw (years)	56.8 (42–71)	56.7 (42–71)	56.7 (41–68)
Physical activity METs (hrs/week)^b^	72.0 (14–149)	72.3 (13–155)	79.2 (18–161)
Dietary variables			
Vitamin D (μg/day)	3.2 (1.2–8.3)	3.2 (1.2–8.6)	3.2 (1.1–8.2)
Calcium (mg/day)	890 (407–1848)	954 (465–1642)	946 (476–1653)
Serum concentrations variables			
25-hydroxyvitamin-D_3,_ nmol/L	42.4 (14.4–79.1)	48.0 (19.2–80.2)	44.2 (20.8–79.7)
**Clinical characteristics, case participants only**			
Age at diagnosis, median (range), years	62 (49–77)		
Time from blood draw to diagnosis	6.3 (0.7–12.9)		
Tumour site, No. (%)			
Esophagus			
Squamous cell carcinoma	73 (15%)		
Adenocarcinoma	74 15%)		
Head and Neck^c^			
Hypopharynx + Larynx	145 (29%)		
Gum + Oral cavity	110 (22%)		
Oropharynx	67 (13%)		
Head and neck other^c^	28 (6%)		

^a^BMI is calculated as weight in kilograms divided by height in meters squared.

^b^Metabolic equivalent intensity values (METs), defined as the ratio of the metabolic rate during an activity to a standard resting metabolic rate of 1.0 (4.184 kJ)·kg^− 1^·hour^−1^

^c^Adenocarcinoma excluded.

**Table 2:** Odds ratios for a doubling in concentration of 25-hydroxyvitamin D_3_ and the risk of cancers of the head and neck and the esophagus.

	No. of controls	Head and neck cancer^a^ (n = 350)	*P* for trend^d^	No. of controls	Esophagus Squamous Cell Carcinoma (n = 73)	*P* for trend^d^	No. of controls	Esophagus Adenocarcinoma (n = 74)	*P* for trend^d^
Minimally adjusted^b^									
Conditional matched control	350	0.55 (0.42–0.72)	*1* × *10*^*−5*^	73	0.78 (0.44–1.37)	*0.39*	74	0.86 (0.44–1.70)	*0.66*
Unconditional all controls combined	940	0.54 (0.44–0.68)	*3* × *10*^*−8*^	940	0.59 (0.38–0.91)	*0.02*	940	0.72 (0.44–1.18)	*0.19*
Fully adjusted^c^									
Conditional matched control	350	0.77 (0.56–1.03)	*0.07*	73	0.83 (0.36–1.94)	*0.67*	74	0.93 (0.39–2.23)	*0.87*
Unconditional all controls combined	940	0.70 (0.56–0.88)	*0.001*	940	0.69 (0.44–1.10)	*0.11*	940	0.74 (0.45–1.23)	*0.26*

^a^Adenocarcinoma excluded.

^b^Conditional adjusted models were assessed by conditional logistic regression, conditioning on matched case set. Unconditional adjusted models were assessed by unconditional logistic regression, adjusted for country, sex, age at recruitment (in 5 year groups) and seasonality.

^c^Fully adjusted models were further adjusted for educational attainment (in 5 groups), smoking status at baseline (never/former/current/missing), cotinine quartiles (based on the distribution among current smokers), alcohol intake at recruitment (g/day), and BMI (in 3 groups).

^d^P for trend assessed by the base 2 logarithm of the circulating levels.

**Table 3:** Odds ratios for a doubling in concentration of 25-hydroxyvitamin D_3_ and the risk of head and neck cancer by tumor sites.

		OR (95% CI)		
	No. of control	Larynx and Hypopharynx cases (n = 144)	Oral cavity and Gum cases (n = 108)	Oropharynx cases (n = 67)
Minimally adjusted^a^	940	0.42 (0.30–0.58)	0.47 (0.33–0.66)	0.79 (0.50–1.24)
*P* for trend^c^		*9* × *10*^*−8*^	*2* × *10*^*−5*^	*0.31*
Fully adjusted^b^	940	0.55 (0.39–0.78)	0.60 (0.42–0.87)	0.92 (0.58–1.45)
*P* for trend^c^		*7* × *10*^*−4*^	*0.005*	*0.71*

^a^Assessed by analysing cancer cases of the head and neck and all controls combined by unconditional logistic regression, adjusting for country, sex, age at recruitment (in 5 year groups) and seasonality.

^b^Fully adjusted models were further adjusted for educational attainment (in 5 groups), smoking status at baseline (never/former/current/missing), cotinine quartiles (based on the distribution among current smokers), alcohol intake at recruitment (g/day), and BMI (in 3 groups).

^c^*P* for trend assessed by the base 2 logarithm of the circulating levels.

## References

[b1] Ferlay J. S. I., Ervik M. . GLOBOCAN 2012 v1.0, Cancer Incidence and Mortality Worldwide: IARC CancerBase No. 11, http://globocan.iarc.fr (2013).

[b2] Tobacco smoke and involuntary smoking. IARC monographs on the evaluation of carcinogenic risks to humans/World Health Organization. International Agency for Research on Cancer 83, 1–1438 (2004). PMC4781536 15285078

[b3] Alcohol consumption and ethyl carbamate. IARC monographs on the evaluation of carcinogenic risks to humans/World Health Organization. International Agency for Research on Cancer 96, 3–1383 (2010). PMC4781168 21735939

[b4] Chaturvedi A. K. . Human papillomavirus and rising oropharyngeal cancer incidence in the United States. Journal of clinical oncology: official journal of the American Society of Clinical Oncology 29, 4294–4301, doi: 10.1200/JCO.2011.36.4596 (2011). 21969503 PMC3221528

[b5] Holick M. F. Vitamin D deficiency. The New England journal of medicine 357, 266–281, doi: 10.1056/NEJMra070553 (2007). 17634462

[b6] Mehta R. G. & Mehta R. R. Vitamin D and cancer. The Journal of nutritional biochemistry 13, 252–264 (2002). 12015155 10.1016/s0955-2863(02)00183-3

[b7] Feldman D., Krishnan A. V., Swami S., Giovannucci E. & Feldman B. J. The role of vitamin D in reducing cancer risk and progression. Nature reviews. Cancer 14, 342–357, doi: 10.1038/nrc3691 (2014). 24705652

[b8] Deeb K. K., Trump D. L. & Johnson C. S. Vitamin D signalling pathways in cancer: potential for anticancer therapeutics. Nature reviews. Cancer 7, 684–700, doi: 10.1038/nrc2196 (2007). 17721433

[b9] Jenab M. . Association between pre-diagnostic circulating vitamin D concentration and risk of colorectal cancer in European populations: a nested case-control study. BMJ 340, b5500, doi: 10.1136/bmj.b5500 (2010). 20093284 PMC2809840

[b10] Ma Y. . Association between vitamin D and risk of colorectal cancer: a systematic review of prospective studies. Journal of clinical oncology: official journal of the American Society of Clinical Oncology 29, 3775–3782, doi: 10.1200/JCO.2011.35.7566 (2011). 21876081

[b11] Skaaby T. . Prospective population-based study of the association between serum 25-hydroxyvitamin-D levels and the incidence of specific types of cancer. Cancer epidemiology, biomarkers & prevention: a publication of the American Association for Cancer Research, cosponsored by the American Society of Preventive Oncology 23, 1220–1229, doi: 10.1158/1055-9965.EPI-14-0007 (2014). 24789846

[b12] Afzal S., Bojesen S. E. & Nordestgaard B. G. Low plasma 25-hydroxyvitamin D and risk of tobacco-related cancer. Clinical chemistry 59, 771–780, doi: 10.1373/clinchem.2012.201939 (2013). 23503722

[b13] Arem H. . Serum 25-hydroxyvitamin D and risk of oropharynx and larynx cancers in Finnish men. Cancer epidemiology, biomarkers & prevention: a publication of the American Association for Cancer Research, cosponsored by the American Society of Preventive Oncology 20, 1178–1184, doi: 10.1158/1055-9965.EPI-11-0153 (2011). PMC3111816 21527582

[b14] Abnet C. C. . Circulating 25-hydroxyvitamin D and risk of esophageal and gastric cancer: Cohort Consortium Vitamin D Pooling Project of Rarer Cancers. American journal of epidemiology 172, 94–106, doi: 10.1093/aje/kwq121 (2010). 20562192 PMC2892544

[b15] Konety B. R. . Effects of vitamin D (calcitriol) on transitional cell carcinoma of the bladder *in vitro* and *in vivo*. The Journal of urology 165, 253–258, doi: 10.1097/00005392-200101000-00074 (2001). 11125420

[b16] Mantell D. J., Owens P. E., Bundred N. J., Mawer E. B. & Canfield A. E. 1 alpha,25-dihydroxyvitamin D(3) inhibits angiogenesis *in vitro* and *in vivo*. Circulation research 87, 214–220 (2000). 10926872 10.1161/01.res.87.3.214

[b17] Meier J. D. . Treatment with 1-alpha,25-dihydroxyvitamin D3 (vitamin D3) to inhibit carcinogenesis in the hamster buccal pouch model. Archives of otolaryngology–head & neck surgery 133, 1149–1152, doi: 10.1001/archotol.133.11.1149 (2007). 18025321

[b18] Mernitz H., Smith D. E., Wood R. J., Russell R. M. & Wang X. D. Inhibition of lung carcinogenesis by 1alpha, 25-dihydroxyvitamin D3 and 9-cis retinoic acid in the A/J mouse model: evidence of retinoid mitigation of vitamin D toxicity. International journal of cancer. Journal international du cancer 120, 1402–1409, doi: 10.1002/ijc.22462 (2007). 17205520

[b19] Nakagawa K., Sasaki Y., Kato S., Kubodera N. & Okano T. 22-Oxa-1alpha,25-dihydroxyvitamin D3 inhibits metastasis and angiogenesis in lung cancer. Carcinogenesis 26, 1044–1054, doi: 10.1093/carcin/bgi049 (2005). 15718253

[b20] Ordonez-Moran P. . Vitamin D and cancer: an update of *in vitro* and *in vivo* data. Frontiers in bioscience: a journal and virtual library 10, 2723–2749 (2005). 15970529 10.2741/1731

[b21] McCullough M. L. . Correlates of circulating 25-hydroxyvitamin D: Cohort Consortium Vitamin D Pooling Project of Rarer Cancers. American journal of epidemiology 172, 21–35, doi: 10.1093/aje/kwq113 (2010). 20562191 PMC2892536

[b22] Jones A. S. . Second primary tumors in patients with head and neck squamous cell carcinoma. Cancer 75, 1343–1353 (1995). 7882285 10.1002/1097-0142(19950315)75:6<1343::aid-cncr2820750617>3.0.co;2-t

[b23] Parkin D. M., Bray F., Ferlay J. & Pisani P. Global cancer statistics, 2002. CA: a cancer journal for clinicians 55, 74–108 (2005). 15761078 10.3322/canjclin.55.2.74

[b24] Pulte D. & Brenner H. Changes in survival in head and neck cancers in the late 20th and early 21st century: a period analysis. The oncologist 15, 994–1001, doi: 10.1634/theoncologist.2009-0289 (2010). 20798198 PMC3228039

[b25] Riboli E. . European Prospective Investigation into Cancer and Nutrition (EPIC): study populations and data collection. Public health nutrition 5, 1113–1124, doi: 10.1079/PHN2002394 (2002). 12639222

[b26] Johansson M. . Circulating biomarkers of one-carbon metabolism in relation to renal cell carcinoma incidence and survival. Journal of the National Cancer Institute 106, doi: 10.1093/jnci/dju327 (2014). PMC4273895 25376861

[b27] Midttun O. & Ueland P. M. Determination of vitamins A, D and E in a small volume of human plasma by a high-throughput method based on liquid chromatography/tandem mass spectrometry. Rapid communications in mass spectrometry: RCM 25, 1942–1948, doi: 10.1002/rcm.5073 (2011). 21698677

[b28] Royston P. & Parmar M. K. Flexible parametric proportional-hazards and proportional-odds models for censored survival data, with application to prognostic modelling and estimation of treatment effects. Statistics in medicine 21, 2175–2197, doi: 10.1002/sim.1203 (2002). 12210632

[b29] R Core Team (2013) R: A language and environment for statistical computing. R Foundation for Statistical Computing, Vienna, Austria. http://www.R-project.org/ (2013).

