# Supplementary material for: Circulating vitamin D in relation to cancer incidence and survival of the head and neck and oesophagus in the EPIC cohort
Source: Sci Rep. 2016 Nov 4;6:36017. doi: 10.1038/srep36017 (PMC5095706; doi:10.1038/srep36017)
Supplement: Supplementary Information [file srep36017-s1.pdf]

Title: Circulating vitamin D in relation to cancer incidence and survival of the head and neck and oesophagus in the EPIC cohort

Authors: Anouar Fanidi[1], David C Muller[1], Øivind Midttun[2], Per Magne Ueland[3,4], Stein Emil Vollset[5,6], Caroline Relton[7], Paolo Vineis[8,9], Elisabete Weiderpass[10], Guri Skeie[10,11], Magritt Brustad[10,11], Domenico Palli[12], Rosario Tumino[13], Sara Grioni[14], Carlotta Sacerdote[15], H.B(as). Bueno-de-Mesquita[16,17], Petra H. Peeters[18], Marie-Christine Boutron-Ruault[19,20,21], Marina Kvaskoff[19,20,21], Claire Cadeau[19,20,21], José María Huerta[22,23], Maria-José Sánchez[22,24], Antonio Agudo[25], Cristina Lasheras[26], J. Ramón Quirós[27], Saioa Chamosa[28], Elio Riboli[8], Ruth C. Travis[29], Heather Ward[8], Neil Murphy[8], Kay-Tee Khaw[30], Antonia Trichopoulou[31,32], Pagona Lagiou[32,33,34], Eleni-Maria Papatesta[31], Heiner Boeing[35], Tilman Kuehn[36], Verena Katzke[36], Annika Steffen[35], Anders Johansson[37], Paul Brennan[1] and Mattias Johansson[1]

1. International Agency for Research on Cancer, Lyon, France
2. Bevital AS, Bergen, Norway
3. Section of Pharmacology, Institute of Medicine, University of Bergen, Bergen, Norway
4. Laboratory of Clinical Biochemistry, Haukeland University Hospital, Bergen, Norway
5. Department of Public Health and Primary Health Care, University of Bergen, Bergen, Norway
6. Division of Epidemiology, Norwegian Institute of Public Health, Bergen, Norway
7. Institute for Ageing and Health, Newcastle University, Newcastle, United Kingdom
8. School of Public Health, Imperial College London, London, United Kingdom
9. HuGeF Foundation, Turin, Italy
10. Department of Community Medicine, Faculty of Health Sciences, University of Tromsø, Tromsø, Norway
11. The Arctic University of Norway, Tromsø, Norway

12. Molecular and Nutritional Epidemiology Unit, Cancer Research and Prevention Institute – ISPO, Florence, Italy
13. Cancer Registry and Histopathology Unit, "Civile M.P.Arezzo" Hospital, ASP Ragusa, Italy
14. Epidemiology and Prevention Unit, Fondazione IRCCS Istituto Nazionale dei Tumori, Milan, Italy
15. CPO-Piemonte and HuGeF Foundation, Torino Turin, Italy
16. National Institute for Public Health and the Environment (RIVM), Bilthoven, The Netherlands
17. Department of Gastroenterology and Hepatology, University Medical Centre, Utrecht, The Netherlands
18. Department of Epidemiology, Julius Center for Health Sciences and Primary Care, University Medical Center, Utrecht, The Netherlands
19. Nutrition, Hormones and Women's Health team, Inserm, Centre for research in Epidemiology and Population Health (CESP), U1018, Villejuif, France
20. Université Paris Sud, UMRS 1018, Villejuif, France
21. Institut Gustave-Roussy (IGR), Villejuif, France
22. Consortium for Biomedical Research in Epidemiology and Public Health (CIBER Epidemiología y Salud Pública-CIBERESP), Madrid, Spain
23. Department of Epidemiology, Murcia Regional Health Council, Murcia, Spain
24. Andalusian School of Public Health, Granada, Spain
25. Catalan Institute of Oncology, L'Hospitalet de Llobregat, Spain
26. Oviedo University, Oviedo, Spain
27. Public Health Directorate Asturias, Oviedo, Spain
28. Public Health Division of Gipuzkoa, BioDonostia Research Institute, Health Department of Basque Region, San Sebastian, Spain
29. Cancer Epidemiology Unit, Nuffield Department of Clinical Medicine, University of Oxford, Oxford, United Kingdom
30. School of Clinical Medicine, University of Cambridge, United Kingdom
31. Hellenic Health Foundation, Athens, Greece

32. WHO Collaborating Center for Food and Nutrition Policies, Department of Hygiene, Epidemiology and Medical Statistics, University of Athens Medical School, Athens, Greece
33. Department of Epidemiology, Harvard School of Public Health, Boston, USA
34. Bureau of Epidemiologic Research, Academy of Athens, Athens, Greece
35. Department of Epidemiology, German Institute of Human Nutrition Potsdam-Rehbruecke, Nuthetal, Germany
36. German Cancer Research Center DKFZ, Heidelberg, Germany
37. Nutritrional Research/Molecular Periodontology Umeå University, Umeå, Sweden

Corresponding author:

Mattias Johansson

Genetic Epidemiology Group

International Agency for Research on Cancer (IARC/WHO)

150 cours Albert Thomas

69008, Lyon, France

Phone: +33 (0) 4 72 73 80 23

Fax: +33 (0) 4 72 73 83 42

E-mail: JohanssonM@iarc.fr

Supplementary Table 1. Linear regression for circulating 25-hydroxyvitamin-D_3 in relation to lifestyle factors, to nutrients and to food groups as assessed by food frequency questionnaires among all controls.

Variable	No.	ratio[a] (95% CI)	P
		**Circulating 25-hydroxyvitamin-D3**	
Never smokers	412	1.00 [reference]	
Former smokers	321	1.09 (1.02 - 1.16)	0.01
Current smokers	191	0.93 (0.87 - 1.00)	0.05
Alcohol intake at recruitment	940	1.02 (0.99 - 1.03)	0.39
Lifetime average alcohol intake per day[b]	803	0.97 (0.96 - 1.04)	0.46
**Following variables adjusted for cotinine (in quartiles) and alcohol at recruitment**			
Vitamin D intake (μg/day)	940	1.03 (0.98 - 1.06)	0.29
Calcium intake (nutrient), mg/day	940	0.97 (0.94 - 1.03)	0.41
Education attainment[c]	908	0.99 (0.97 - 1.02)	0.39
Having employment[d]	785	0.95 (0.89 - 1.01)	0.08
Total phisical activity[e]	852	1.02 (0.98 - 1.05)	0.12
Physical activity at work[f]	560	1.04 (1.00 - 1.09)	0.07
Body mass index[g]	940	0.95 (0.92 - 0.98)	0.006

[a] Ratios of circulating concentrations were calculated as 2 raised to the power of the beta estimates from linear regression models with log2- serum concentrations as response. The linear regression models were adjusted age, sex, country and as indicated adjusted for cotinine (in quartiles) and alcohol at recruitment. The base 2 logarithm of each nutrient or food group was included as covariate in the linear regression models. The concentration ratio can thus be interpreted as the average relative change in serum levels that would be expected for a doubling in dietary intake.

[b] coded 0/1/2/3/4/5/6/ ~ alcohol[g/day]=0 / 0.1-6 / 6.1-12 / 12.1-24 / 24.1-60 / 60.1-96 in men or >60 in women / >90 in men. Adjusted for case-control status, age, sex, country and cotinine (in quartiles) among all controls.

[c] coded 1/2/3/4 ~ Primary school completed / Technical/professional school / Secondary school / Longer education (incl. University deg.)

[d] coded 0/1 ~ unemployed / employed

[e] coded 1/2/3/4 ~ inactive / moderately inactive / moderately active / active

[f] coded 1/2/3/4 ~ Sedentary occupation / Standing occupation / Manual work /Heavy manual work

[g] coded 1/2/3/4 ~ BMI [kg/m2] <25 / 25-24.99/ >= 30

Supplementary Figure 1. Stratified odds ratios (95% CI) for a doubling in concentration of 25-hydroxyvitamin-D_3 for head and neck cancer[a]

Covariate	Cases	Controls	OR[b]	95%CI	OR[c]	95%CI
**Overall log2 - 25-hydroxyvitamin D_3**	350	940	0.54	0.44-0.68	0.70	0.56-0.88
**Country (p-heterogeneity= 0.19)**					**(p-heterogeneity= 0.17)**	
France/Italy/Spain/Greece	147	349	0.44	0.31-0.62	0.59	0.40-0.87
Uk/Germany/Netherlands	184	520	0.65	0.49-0.87	0.77	0.57-1.06
Sweeden/Norway	19	71	0.37	0.09-1.60	0.05	0.01-0.99
**Histology (p-heterogeneity= 0.08)**					**(p-heterogeneity= 0.28)**	
Hypopharynx and Larynx	145	940	0.42	0.30-0.58	0.56	0.39-0.79
Gum and Oral cavity	110	940	0.46	0.33-0.66	0.61	0.42-0.87
Oropharynx	67	940	0.79	0.50-1.24	0.89	0.56-1.41
**Smoking status (p-heterogeneity= 0.02)**					**(p-heterogeneity= 0.07)**	
Never smokers	64	412	1.21	0.72-2.03	1.22	0.69-2.08
Former smokers	88	321	0.65	0.42-1.01	0.64	0.41-1.01
Current smokers	194	191	0.47	0.33-0.66	0.55	0.39-0.80
**Age at diagnosis, years (p-heterogeneity= 0.98)**					**(p-heterogeneity= 0.99)**	
38-49.9	30	78	0.52	0.26-1.05	0.67	0.27-1.67
50-59.9	116	259	0.49	0.33-0.73	0.71	0.46-1.09
60-69.9	156	438	0.56	0.40-0.79	0.66	0.46-0.96
70-85.3	48	163	0.53	0.29-0.98	0.70	0.34-1.41
**Educational attainment (p-heterogeneity= 0.42)**					**(p-heterogeneity= 0.32)**	
Primary school	148	363	0.49	0.35-0.70	0.66	0.46-0.95
Higher than primary school	189	545	0.59	0.44-0.79	0.77	0.55-1.06
**Time from blood draw to diagnosis, months (p-heterogeneity= 0.47)**					**(p-heterogeneity= 0.79)**	
1-35	92	224	0.50	0.32-0.79	0.62	0.37-1.05
36-72	77	209	0.73	0.46-1.15	0.86	0.52-1.41
73-108	96	243	0.52	0.34-0.79	0.70	0.43-1.14
109-151	85	260	0.41	0.26-0.66	0.54	0.31-0.92
**Body mass index (p-heterogeneity= 0.07)**					**(p-heterogeneity= 0.18)**	
<25	136	376	0.45	0.32-0.63	0.59	0.40-0.86
25-29.9	151	418	0.55	0.39-0.77	0.81	0.56-1.18
>=30	63	146	1.08	0.56-2.11	1.19	0.59-2.40
**Sex (p-heterogeneity= 0.08)**					**(p-heterogeneity= 0.48)**	
Men	248	574	0.47	0.36-0.62	0.66	0.49-0.90
Women	102	366	0.71	0.50-1.02	0.79	0.53-1.18
**Alcohol intake at recruitment , g/day (p-heterogeneity= 0.15)**					**(p-heterogeneity= 0.22))**	
=0	59	112	0.91	0.50-1.66	1.75	0.79-3.88
0-6	83	323	0.71	0.45-1.13	0.76	0.47-1.22
6-12	31	129	0.53	0.24-1.21	0.72	0.28-1.87
12-24	51	170	0.35	0.19-0.65	0.48	0.24-0.97
>24	125	206	0.42	0.29-0.63	0.57	0.36-0.90

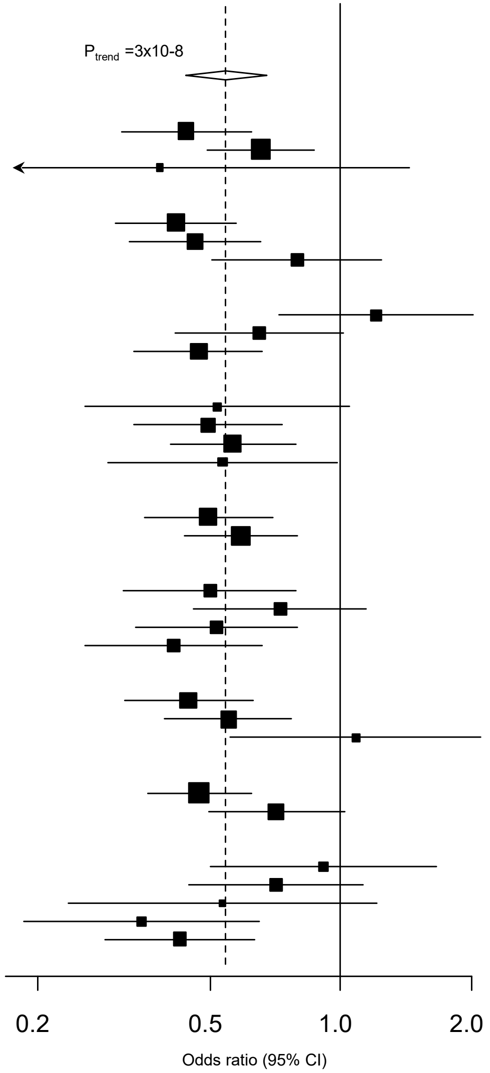

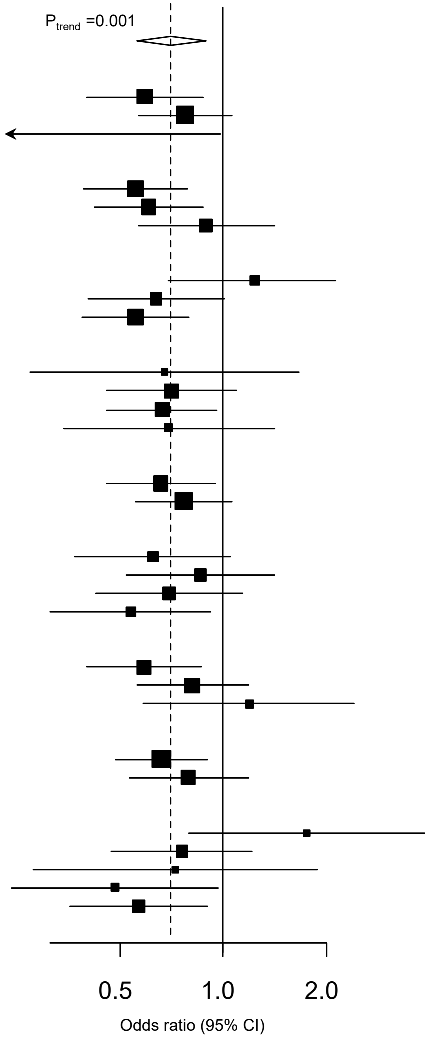

[a] Head and neck cancer cases (adenocarcinoma excluded) and controls included in each stratified analysis (control group 2 was included).
[b] Assessed by unconditional logistic regression by including the base 2 logarithm of circulating concentrations (ORs indicate relative risks of a doubling in plasma concentrations), for seasonality and where relevant for age (in 5-year categories), sex and country.
[c] Further adjusted for educational attainment (in 5 groups), smoking status (never/former/current/missing), circulating cotinine (quartiles defined among the current smokers), alcohol intake at recruitment (g/day), and BMI (in 3 groups)where relevant
The black dots indicate the ORs and the horizontal lines indicate the 95% confidence intervals.
P heterogeneity indicates results of chi-square test assessing the null hypothesis of ORs being the identical.

Supplementary Figure 2. Forest plot showing stratified hazard ratios of all-cause mortality for head and neck cancer cases[a] for a doubling in concentration of 25-hydroxyvitamin-D_3.

study group	Events	Cases	HR[b]	95%CI
**Overall log2 - 25-hydroxyvitamin D3**	145	350	0.73	0.55-0.97
**By Country (p-heterogeneity= 0.32)**				
France/Italy/Spain/Greece	55	147	0.92	0.57-1.49
Uk/Germany/Netherlands	83	184	0.69	0.51-0.94
Sweeden/Norway	7	19	inf	0.00-inf
**By smoking status (p-heterogeneity= 0.51)**				
Never smokers	19	64	0.79	0.15-4.09
Former smokers	34	88	0.94	0.55-1.60
Current smokers	89	194	0.65	0.47-0.90
**By age at diagnosis (p-heterogeneity= 0.39)**				
38-49.9 years	13	30	1.46	0.40-5.30
50-59.9 years	43	116	0.70	0.41-1.18
60-69.9 years	65	156	0.65	0.43-0.98
70-85.3 years	24	48	0.87	0.35-2.12
**By educational attainment (p-heterogeneity= 0.16)**				
Primary school	66	148	0.94	0.64-1.38
Higher than primary school	75	189	0.64	0.44-0.93
**By gender (p-heterogeneity= 0.15)**				
Men	113	248	0.86	0.63-1.16
Women	32	102	0.49	0.24-0.99
**By alcohol intake at recruitment (p-heterogeneity= 0.15)**				
=5.9 g/day	60	142	0.78	0.50-1.24
6-17.9 g/day	19	58	0.19	0.05-0.74
18-29.9 g/day	10	43	0.58	0.42-0.80
>=30 g/day	56	107	0.78	0.53-1.14

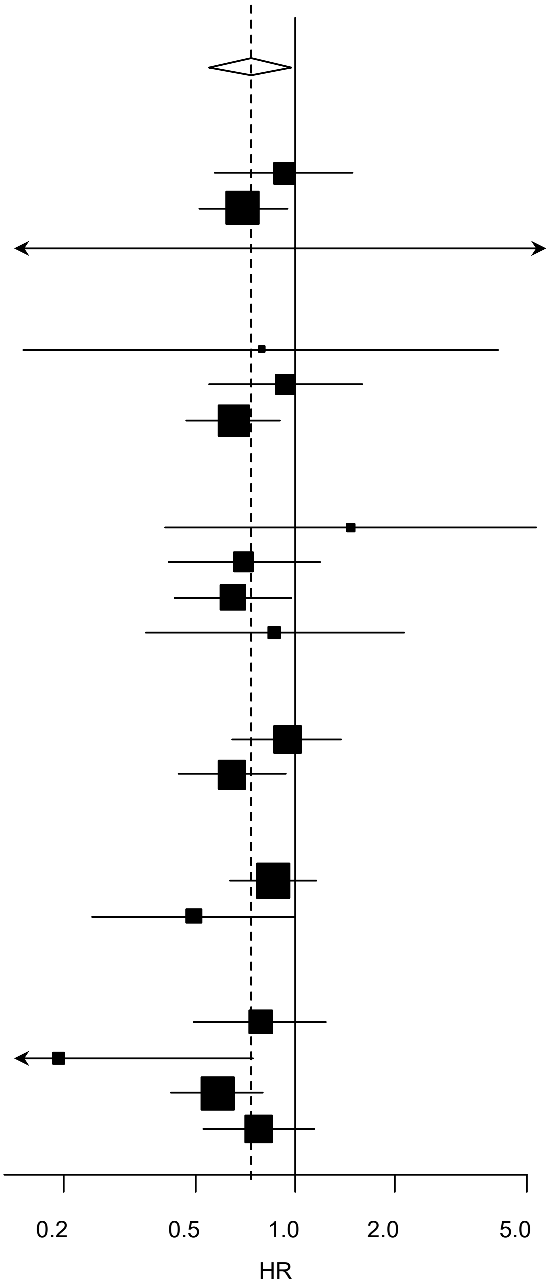

[a] Head and neck cancer cases (adenocarcinoma excluded) included in each stratified analysis.
[b] Assessed by analysing HNC cases by Cox-proportional hazards regression by including the base 2 logarithm of plasma analyte concentrations, adjusting for seasonality and where relevant for age at diagnosis (in categories), sex, country, educational attainment (in 5 groups), smoking status (never/former/current/missing), circulating cotinine (quartiles defined among the current smokers) alcohol intake at recruitment (g/day), and BMI (in 3 groups); the black dots indicate the HRs and the horizontal lines indicate the 95% confidence intervals.
P heterogeneity indicates results of chi-square test assessing the null hypothesis of HRs being the identical.
